# Job preferences for healthcare administration students in China: A discrete choice experiment

**DOI:** 10.1371/journal.pone.0211345

**Published:** 2019-01-25

**Authors:** Shimeng Liu, Shunping Li, Yujia Li, Haipeng Wang, Jingjing Zhao, Gang Chen

**Affiliations:** 1 School of Health Care Management, Shandong University, Jinan, China; 2 NHC Key Laboratory of Health Economics and Policy Research (Shandong University), Jinan, China; 3 School of Public Health, Shandong University, Jinan, China; 4 College of Medicine and Public Health, Flinders University, Adelaide, Australia; Charles P. Darby Children’s Research Institute, 173 Ashley Avenue, Charleston, SC 29425, USA, UNITED STATES

## Abstract

**Background:**

There is a deficiency of healthcare administrators in China as compared with other countries; furthermore, the distribution is unequal. To inform an effective policy intervention, it is crucial to understand healthcare administration students’ career decision-making. This study aims to investigate the undergraduate students’ stated preferences when choosing a job.

**Methods:**

A discrete choice experiment (DCE) was conducted among a population-based multistage sample of 668 final year undergraduate healthcare administration students during April to June 2017 in eight universities of China to elicit their job preferences. Attributes include location, monthly income, *bianzhi* (which refers to the established posts and can be loosely regarded as state administrative staffing), training and career development opportunity, working environment and workload. Conditional and mixed logit models were used to analyze the relative importance of job attributes.

**Results:**

All six attributes were statistically significant with the expected sign and demonstrated the existence of preference heterogeneity. Monthly income, workload and working environment were of most concern to healthcare administration students when deciding their future. Among the presented attributes *bianzhi* was of the least concern. Sub-group analysis showed that students who have an urban background and/or with higher annual family incomes were willing to pay more for working in the city. In addition, students from western and middle universities valued *bianzhi* higher than students from eastern universities.

**Conclusions:**

This is the first study focusing on the career decision-making of Chinese healthcare administration students at a critical career decision-making point. Both monetary and non-monetary interventions could be considered by policy-makers to attract students to work in health institutions, especially in rural and remote health institutions in China. There exists preference heterogeneity on healthcare administration students’ job preferences, which should also be taken into account in developing more effective policy incentive packages.

## Introduction

Effective health reforms will necessarily deal with the three major resource inputs of any national health system: financial, physical and human resources [1]. Undoubtedly, it is more difficult to allocate the limited human resources due to the dynamically changing nature of this resource [2]. Achieving equity in health is an important aspect of social fairness, whereas one of the biggest challenges is to achieve equity in health workforce distribution [3–6]. Although the World Health Organization has recommended several policy interventions, such as recruiting students with a rural background and embedding a rural course so that students would become more familiar with remote conditions and more likely to return to rural areas after graduation [6], the unevenly distributed health workforce remains a significant issue in both developed and developing countries. In China, human resources for health were considered as the least mapped and analyzed [1]. The distribution of health professionals is largely determined by the market instead of the government because health professionals have the right to practice wherever they choose based on their own preferences and the availability of positions [7].

The fundamental objectives of the healthcare management profession are to maintain or enhance the overall quality of life [8]. In Europe and in the United States, health management education and the role of health managers are patterned and consistent with how the country’s healthcare system is organized, managed, and financed [9]. The evidence worldwide is that health management is not as mature a profession as medicine, law, and nursing, nor do health management programs generally have much independent academic autonomy within their universities [9]. It has been described as a "hidden" health profession [10] because of the relatively low-profile role managers take in health systems, in comparison to direct-care professions such as nursing and medicine. However the visibility of the management profession within healthcare has been rising in recent years, largely due to the widespread problems that developed countries are having in balancing cost, access, and quality in their hospitals and health systems [11].

In China, healthcare administration is a subject that explores the development rule of health service enterprise, the task of which is to study the theory and methods of health management, health policy fit for the situation of China, organization management or work method in step with the correct policy, and the experience of health management from the countries all over the world [12]. The undergraduate training in China differs with other developed counties in terms of the curriculum, education style and objective of the program [13, 14]. Graduates from the healthcare administration undergraduate program in China are equipped to work as administrative staff in various health institutions, such as hospitals, health inspection institutes, and the center for disease prevention and control [15–18].

Although the Chinese government has launched major reforms of the healthcare system to attract health professionals to work in rural areas, the unequal allocation of human resources for health has worsened [19, 20]. The number of healthcare administrators in China is deficient compared with other countries [18] and above 96% of the final year undergraduates healthcare administration students prefer to find jobs through the talent market and concentrate in large cities; only less than 4% plan to devote themselves to the rural areas [21]. One survey conducted by Fudan University in 2010 indicated nearly one third of their final year undergraduate healthcare administration students were employed by enterprises which are unrelated to any type of health field or science [15]. Along with the current healthcare reforms in China, effective policies will be needed to develop and manage its health workforce.

Understanding health professionals’ preferences on job characteristics is crucial for designing an effective policy intervention. Worldwide, there is an increasing amount of literature using discrete choice experiments (DCEs) to examine factors that influencing health professionals’ career decision-making [22–26]. DCE is a quantitative technique for understanding individual preferences and it has been extensively applied in health care research to address different policy and research issues [25, 27, 28]. It has also been found that stated preferences derived from a DCE can adequately predict actual behavior in a public health setting [29].

This study aims to elicit job preferences of final year undergraduate healthcare administration students in China (whose career-related decisions are crucial to the development of the health service in the future), and it represents the first DCE in this context. Results from this study will provide guidance on career choice and planning for students and inform policy-makers to develop more effective policies for the attraction and retention of healthcare administration students to health institutions, especially to rural and remote health institutions.

## Materials and methods

### Sampling

This study used a multistage sampling design. First, seven provinces were selected according to their economic development level to represent eastern (Heilongjiang, Liaoning, Beijing and Shandong), middle (Shanxi) and western (Gansu, Ningxia) China. Next, one or two universities were chosen based on the representativeness of their socio-economic status and the development of the healthcare administration subject. Finally, the participants of this study were recruited from university under the direct affiliation of the central ministries and commissions (Lanzhou University), universities of traditional Chinese medicine (Beijing University of Chinese Medicine, Shandong University of Traditional Chinese Medicine) and provincial independent medical universities (Harbin Medical University, Dalian Medical University, Weifang Medical University, Shanxi Medical University and Ningxia Medical University). We aimed to recruit a minimum of 100 respondents in eastern, middle and western China [30–32]. Consequently, one to three classes in each university were randomly selected depending on the number of students in each class.

### Discrete choice experiment

The methodology of DCEs is presented as an example of the stated preference method (SPM) that allows for measurement of health workers’ preferences for a combination of job attributes, and quantitatively predicts the job uptake given a set of job characteristics [33]. It has become a commonly used instrument in health economics [34]. Random utility theory [30] provides the theoretical foundation for DCEs: a discrete choice is offered and participants choose the option with the highest utility among the alternatives presented. In the context of this study, it assumes that a job scenario can be described by a series of attributes and their corresponding levels [35]. The DCE design and analysis was conducted following the International Society For Pharmacoeconomics and Outcomes Research (ISPOR) good practice principals outlined by Bridges et al. [36].

The first step to design DCEs is to identify the attributes and corresponding levels. Two main concerns should be taken into account when selecting attributes and attribute levels: 1) they should be relevant for policy purposes; and 2) they have to be meaningful and important to respondents [37]. There is a gold standard to use qualitative research methods (focus groups discussion or/and in-depth interviews) to identify and define the attributes and attribute levels [38]. Initially eight attributes which have been commonly adopted and suitable for the Chinese health system were identified through a literature review [22, 39–41], including working location, monthly income, *bianzhi* (which refers to the established posts and can be loosely regarded as state administrative staffing) [42], training and career development opportunity, housing (housing offered or not), hospital type (determined by the current provision situation within China: primary hospital, secondary hospital and tertiary hospital), management style (supportive or unsupportive workplace and management) and workload. In-depth interviews were then conducted with eight healthcare administration students from two different universities (Shandong University of Traditional Chinese Medicine and Shanxi Medical University). During the interviews, participants were asked to comment on a candidate list of job attributes which were extracted from the literature review and to indicate any additional candidate attributes that were omitted from the literature review. It was suggested that the attribute “hospital type” and “housing” be removed. In addition, two focus group discussions were conducted among fourteen final year healthcare administration students from Lanzhou University and Weifang Medical University. During the focus group discussion, participants were asked to discuss the remaining six attributes and their levels until they reached a consensus for the final version of attributes and levels. They were also asked to provide other attributes that they thought were important but were not in the list. As a result, the “management style” was removed and the “working environment” was further added as a new attribute. In sum, the final attributes and their levels (Table 1) were determined on the basis of the literature review, in-depth interview, focus group discussion as well as discussion with a senior health economist who is an expert on DCEs.

**Table 1:** DCE attributes and levels for final year healthcare administration students in China.

Attributes	Definition	Attribute levels
**Location**	Location refers to working in health institutions of different regions.	Township or rural
		County
		City
**Monthly income**	Including salary, bonus and welfare benefits.	2000 CNY
		5000 CNY
		8000 CNY
***Bianzhi***	*Bianzhi* refers to the authorized number of personnel (the number of established posts) in a party or government administrative organ.	None
		Offer
**Training and career development opportunity**	Training and career development opportunity represents the chances of getting professional promotion and the opportunity to attend short-term courses to develop professional skills.	Insufficient
		Average
		Sufficient
**Working environment**	Working environment refers to management support, the relationship between superior and subordinate, amenities (such as regular bus, canteen and lounge), high-risk work environments and availability of equipment.	Poor
		Common
		Superior
**Workload**	Including the workload in the daytime (whether they have enough time to complete duties) and the conditions of working overtime.	Heavy
		Normal
		Light

According to the Organisation for Economic Co-operation and Development (OECD) data (https://data.oecd.org/conversion/exchange-rates.htm), the average annual exchange rate between US$ and CNY in 2017 was: US$1 = CNY 6.759.

Once the attributes and attribute levels are defined, they were combined into a set of carefully selected scenarios (choice sets). Out of six determined DCE attributes, five attributes had three levels, one attribute had two levels. A full factorial design will generate 486 (3^5^×2^1^) possible scenarios and a total of 117,855 possible pair wise choices ((486×485)/2). A D-efficient (*D*_*z*_-error, i.e. zero priors assumed for all variables) design, for main effects only, was developed using Ngene 1.1.2 (Choice-Metrics, Sydney, Australia), which yielded 24 choice sets that were further divided into two blocks so as to minimise participants’ cognitive burden. Within each version, a single choice set was duplicated to examine the internal consistency of participants.

A pair wise binary two-stage response DCE design was used to maximise the information gained from the participants [43]. In the first stage, each participant made a choice between two alternative job scenarios. Secondly, participants were asked a follow up question as to whether in real life they would be willing to participate in their preferred job from stage one (see Table 2 for an example choice set, and another example in Chinese in the S1 Fig). The above two questions were combined together for analysis to take into account an ‘opt out’ option [44].

**Table 2:** Example combination of choice: Which of these jobs would you prefer?

Attributes	Job Scenario 1	Job Scenario 2
Location	Township or rural	City
Monthly income	2000 CNY	5000 CNY
*Bianzhi*	Offer	None
Training and career development opportunity	Sufficient	Insufficient
Working environment	Poor	Superior
Workload	Light	Normal
Which of these jobs would you prefer?		
Will you actually take up the job you chose if it was offered to you?	Yes	No

### Survey and data collection

In addition to the DCE questions, the hard-copy questionnaire also contains questions related to participants’ socio-demographic characteristics, career planning and annual family income. The full questionnaire was piloted among forty final year healthcare administration students in Weifang Medical University before data was collected between April and July, 2017, aiming to examine the comprehensibility, acceptability, and validity of the questionnaire. The timing of the survey was chosen at that time period because students had finished specialty practice and were considering job opportunities but had not yet made their placement decisions [22]. The survey was conducted in a classroom or dormitory setting. The meaning of the survey as well as the instructions on the DCEs was explained in detail by one or two researchers. Then students filled in the questionnaire by themselves and all responses were anonymous. The process of administering the questionnaire took about 20 to 30 minutes on average and all completed questionnaires were returned directly to the investigators.

All the participants provided informed verbal consent before completing the questionnaire. A detailed explanatory statement was given to respondents describing the study, which highlighted that their participation was voluntary and no identifiable personal data would be collected. It is an anonymous survey so that we did not require a written consent. As explained above, respondents were fully aware of the aim and task of the study before they provided verbal consent to participate this study. A return questionnaire also indicates the implied consent. The implied consent through a return questionnaire is commonly adopted in the anonymous (online) survey. Ethical approval (Reference No.20170301) was obtained both for the consent procedure and for the study as a whole from the Ethics Review Board of the School of Preventive Medicine, Shandong University, and the research adhered to the tenets of the Declaration of Helsinki.

### Data analysis

Data were double-entered into EpiData 3.1 (EpiData Association, Odense, Denmark) and transferred to Stata 12.1 (StataCorp LP, College Station, Texas, USA) for processing and analysis. Descriptive statistics were reported for participants’ socio-demographic characteristics. The data from the DCE were analyzed within a random utility theory framework. The utility function can be specified as follows:

$$U_{ijt}=X_{ijt}^{\prime }\beta_{i}+\varepsilon_{ijt}$$

where U_ijt_ is the utility individual *I* derives from choosing alternative *j* in choice scenario *t*, *X* is a vector of observed attributes (i.e., the job preferences attributes and corresponding levels), *β* is a vector of coefficients reflecting the desirability of the attributes, and ε_ijt_ is an error term. Two econometric approaches were used to estimate this utility function, including the classical conditional logit model and a mixed logit model that could be used to capture potential unobservable preference heterogeneity [22, 45]. In the mixed logit model, the desirability of attributes constitutes a vector of average preferences of the population for each attribute (*β*) and the individual’s specific preference components (*η*) (i.e., *β*_*i*_ = *β* + *η*_*i*_), whereas in the conditional logit model, only average preferences are estimated (i.e., *β*_*i*_ = *β*). Conditional and mixed logit regression models were compared using the Akaike Information Criterion (AIC) and Bayesian Information Criterion (BIC), which is commonly used for model selection in random utility framework [30, 46, 47].

Although most previous studies specify the coefficient for monetary attribute in choice models to be fixed, it is often unrealistic to assume that all participants have the same preferences regarding the monthly income of a job position [48]. In our study, all attributes were dummy coded and specified as having a random component, except for monthly income which was specified as a continuous variable in the models to facilitate the calculation of willingness to pay (WTP), that is, the relative monetary value that students place on various aspect of the job options [49]. Through calculating the ratios of the coefficients between each attribute level and the salary attribute, the marginal rate of substitution or WTP was calculated ($-\frac{\beta q}{\beta m}$ where *β*_*m*_ is the salary coefficient and *β*_*q*_ is the coefficient for attribute *q*) [50]. The positive and negative results indicate theoretically to what extent the participants would be willing to pay/to be compensated for an attribute level. The 95% confidence intervals were estimated using the Krinsky Robb (parametric bootstrap) method [51]. Finally, we also conducted a simulation study to understand to what extent the probability of choosing a given post changes as the levels of the attributes are changed [52].

## Results

### Respondents

The response rate to the questionnaire was 668 (95.2%) out of 702 for final year healthcare administration students. Of these, 22 (3.3%) participants who did not complete the majority of DCE tasks were excluded from the analysis. For internal consistency, a choice test based on duplicated choice tasks among the remaining 646 participants resulted in 69 (10.7%) participants failing the test, and there were no statistically significant differences on demographic characteristics between those who failed versus who passed the test (Table 3). For those participants who passed the consistency test, they (n = 577) had a mean age of 22.2 years (standard deviation, SD = 1.07), most (74.5%) were female and only 31.9% participants came from urban areas. Over two-thirds students were not the single-child within their families. Around 39.7% of them prefer the job market and 46.8% prefer to further their study after graduation.

**Table 3:** Demographic characteristics of final year healthcare administration students.

Characteristics of Respondents	Full sample: n = 646		Analysis sample: n = 577 (who passed the consistency test)		Excluded sample: n = 69 (who failed the consistency test)		χ2 (*P*-value)
	n	%	n	%	n	%	
Age(year), Mean ± SD	22.2	± 1.09	22.2	± 1.07	22.3	± 1.28	
**Gender**							3.649(0.056)
Male	172	26.6%	147	25.5%	25	36.2%	
Female	474	73.4%	430	74.5%	44	63.8%	
**Birthplace**							0.364(0.834)
Urban	208	32.2%	184	31.9%	24	34.8%	
County	127	19.7%	115	19.9%	12	17.4%	
Rural	311	48.1%	278	48.2%	33	47.8%	
**Single child**							0.005(0.942)
Yes	246	38.1%	220	38.1%	26	37.7%	
No	400	61.9%	357	61.9%	43	62.3%	
**Monthly consumption**							0.759(0.859)
< 800 CNY	98	15.2%	88	15.3%	10	14.5%	
800–1500 CNY	404	62.5%	363	62.9%	41	59.4%	
1500–2500 CNY	116	18.0%	102	17.7%	14	20.3%	
> 2500 CNY	28	4.3%	24	4.2%	4	5.8%	
**Annual family income**							2.193(0.700)
< 30000 CNY	137	21.2%	121	21.0%	16	23.2%	
30000–50000 CNY	205	31.7%	182	31.5%	23	33.3%	
50000–70000 CNY	108	16.7%	100	17.3%	8	11.6%	
70000–90000 CNY	69	10.7%	63	10.9%	6	8.7%	
> 90000 CNY	127	19.7%	111	19.2%	16	23.2%	
**Career planning**							5.068(0.079)
Do the health related job	259	40.1%	229	39.7%	30	43.5%	
Further study	294	45.5%	270	46.8%	24	34.8%	
Others	93	14.4%	78	13.5%	15	21.7%	

US$1 = CNY 6.759.

### DCE estimates

The DCE results based on the full sample are reported in Table 4 and S1 Table. It can be seen that the main findings are similar regardless of whether those participants who did not pass the consistency test are included or excluded. As such, the following discussions are based on those who passed the consistency test only. Meanwhile, a sensitivity analysis was conducted by including participants who failed the test. The AIC and BIC values further suggested that the mixed logit estimates were preferable to the conditional logit estimates for the analysis sample and the results from mixed logit model were not substantially different from the conditional logit model. As such, we only report the preferred mixed logit estimates in Table 4. The conditional logit estimates are presented in S2 Table.

**Table 4:** Mixed logit estimates and WTP (n = 577).

Attribute levels	β (SE)	*P*-value	SD (SE)	*P*-value	WTP(CNY)	95% CI	
**ASC (opt-out)**	4.727(0.174)	<0.001	1.970(0.112)	<0.001			
**Location: Township or rural (ref)**							
County	0.427(0.060)	<0.001	0.061(0.362)	0.866	897.347	646.379	1153.207
City	1.006(0.075)	<0.001	1.018(0.080)	<0.001	2112.949	1798.462	2432.324
***Bianzhi*: None (ref)**							
Offer	0.734(0.059)	<0.001	0.831(0.070)	<0.001	1542.568	1312.189	1779.948
**Training and career development opportunity: Insufficient (ref)**							
Average	0.025(0.062)	0.689	0.391(0.126)	0.002	52.019	-206.238	306.783
Sufficient	0.846(0.066)	<0.001	0.563(0.102)	<0.001	1776.968	1500.845	2062.224
**Working environment: Poor (ref)**							
Common	0.965(0.065)	<0.001	0.029(0.103)	0.776	2026.354	1758.532	2296.157
Superior	1.141(0.064)	<0.001	0.262(0.153)	0.087	2397.535	2129.055	2669.811
**Workload: Heavy (ref)**							
Normal	0.953(0.062)	<0.001	0.045(0.181)	0.802	2001.856	1753.245	2261.014
Light	1.161(0.067)	<0.001	0.042(0.159)	0.790	2439.185	2182.194	2705.497
**Monthly income**	0.000476(0.000015)	<0.001	0.000142(0.000017)	<0.001			
AIC	10534.32						
BIC	10709.03						
Log likelihood	-5245.159						
Respondents, n	577						
Observations, n	20772						

β: The coefficients (β) represents the mean relative utility of each attribute conditional on other attributes in a choice set where larger values indicate greater utility and more preferred attributes; ASC (opt-out): Alternative Specific Constant for opt-out; AIC: Akaike Information Criterion; BIC: Bayesian Information Criterion; SD: Standard Deviation estimates reflect preference heterogeneity in the students, a possible indication of unmeasured factors influencing the strength and direction of preference; 95% CI = 95% Confidence Interval; SE: Standard Error.

Firstly, the statistical significance of at least one level of each attribute indicates that all key characteristics identified in the DCE design stage played a significant role in job choice. Secondly, unobservable preference heterogeneity (as reflected in the estimated standard deviations of the mean coefficients) existed for four out of six attributes, with the two attributes having homogeneous preference being working environment and workload. Thirdly, the positive coefficients indicate that an improvement in the characteristic was associated with an increased preference for a job position. Finally, on average participants in this study indicated a negative preference to take a job (as indicated by the significantly positive coefficient attached the alternative-specific constant, ‘ASC (opt-out)’).

### Willingness to pay

The results of the WTP calculation are also shown in Table 4 and are used for relative comparisons. Compared to the reference levels for each attribute, workload and working environment were most strongly associated with job preferences. For example, students were willing to pay 2439 CNY and 2398 CNY to obtain a job position with light workload and superior working environment rather than heavy workload and poor working environment respectively. In terms of offering *bianzhi* or not, they were willing to pay about 1543 CNY to get it. The results of selective sub-group analyses were presented in Tables 5–9. For the subgroups, all six attributes remained statistically significant in influencing preferences. Focusing on the WTP estimates, it can be seen that students from single-child family and students who have an urban background and/or with higher annual family incomes were willing to pay more for working in the city. Students from western and middle universities would be willing to pay 749 CNY and 903 CNY more for a job with *bianzhi* than students from eastern universities respectively. There were no significant differences between male and female students, and those who opt to further study versus who opt to get a job.

**Table 5:** Subgroup analyses: Location.

Attribute levels	Urban background				Rural or county background			
	β (SE)	*P*-value	SD (SE)	*P*-value	β (SE)	*P*-value	SD (SE)	*P*-value
**ASC (opt-out)**	6.150(0.337)	<0.001	1.833(0.168)	<0.001	4.239(0.200)	<0.001	1.819(0.134)	<0.001
**Location: Township or rural (ref)**								
County	0.462(0.137)	0.001	0.515(0.214)	0.016	0.417(0.070)	<0.001	0.096(0.195)	0.624
City	2.120(0.163)	<0.001	1.213(0.146)	<0.001	0.566(0.078)	<0.001	0.646(0.103)	<0.001
***Bianzhi*: None (ref)**								
Offer	0.597(0.114)	<0.001	0.919(0.124)	<0.001	0.790(0.070)	<0.001	0.767(0.080)	<0.001
**Training and career development opportunity: Insufficient (ref)**								
Average	-0.060(0.129)	0.642	0.528(0.179)	0.003	0.041(0.072)	0.567	0.390(0.146)	0.008
Sufficient	0.799(0.121)	<0.001	0.391(0.249)	0.117	0.846(0.077)	<0.001	0.566(0.115)	<0.001
**Working environment: Poor (ref)**								
Common	1.008(0.129)	<0.001	0.050(0.167)	0.766	0.992(0.077)	<0.001	0.036(0.148)	0.810
Superior	1.185(0.126)	<0.001	0.179(0.312)	0.566	1.124(0.076)	<0.001	0.327(0.141)	0.020
**Workload: Heavy (ref)**								
Normal	0.527(0.120)	<0.001	0.027(0.179)	0.881	1.131(0.074)	<0.001	0.009(0.212)	0.966
Light	1.094(0.130)	<0.001	0.125(0.322)	0.699	1.190(0.079)	<0.001	0.073(0.159)	0.647
**Monthly income**	0.000517 (0.000029)	<0.001	0.000115 (0.000030)	<0.001	0.000471 (0.000019)	<0.001	0.000154 (0.000018)	<0.001
Log likelihood	-1609.050				-3522.915			
Respondents, n	184				393			
Observations, n	6624				14148			

ASC (opt-out): Alternative Specific Constant for opt-out; SD: Standard Deviation; SE: Standard Error.

**Table 6:** Subgroup analyses: Family income.

Attribute levels	Annual family income: > 50000 CNY				Annual family income: ≤ 50000 CNY			
	β (SE)	*P*-value	SD (SE)	*P*-value	β (SE)	*P*-value	SD (SE)	*P*-value
**ASC (opt-out)**	6.071(0.303)	<0.001	2.172(0.168)	<0.001	3.899(0.207)	<0.001	1.694(0.153)	<0.001
**Location: Township or rural (ref)**								
County	0.601(0.107)	<0.001	0.524(0.198)	0.008	0.305(0.077)	<0.001	0.022(0.177)	0.900
City	1.718(0.130)	<0.001	1.197(0.122)	<0.001	0.518(0.089)	<0.001	0.738(0.106)	<0.001
***Bianzhi*: None (ref)**								
Offer	0.761(0.096)	<0.001	0.961(0.110)	<0.001	0.687(0.077)	<0.001	0.772(0.084)	<0.001
**Training and career development opportunity: Insufficient (ref)**								
Average	0.057(0.105)	0.587	0.625(0.143)	<0.001	0.032(0.078)	0.683	0.303(0.186)	0.104
Sufficient	1.081(0.106)	<0.001	0.518(0.159)	0.001	0.684(0.085)	<0.001	0.566(0.125)	<0.001
**Working environment: Poor (ref)**								
Common	1.042(0.105)	<0.001	0.072(0.196)	0.712	0.899(0.084)	<0.001	0.025(0.128)	0.844
Superior	1.113(0.105)	<0.001	0.502(0.150)	0.001	1.172(0.082)	<0.001	0.048(0.200)	0.811
**Workload: Heavy (ref)**								
Normal	0.943(0.101)	<0.001	0.058(0.211)	0.784	0.979(0.079)	<0.001	0.001(0.296)	0.998
Light	1.295(0.109)	<0.001	0.308(0.169)	0.069	1.087(0.085)	<0.001	0.048(0.168)	0.774
**Monthly income**	0.000513 (0.000025)	<0.001	0.000120 (0.000028)	<0.001	0.000462 (0.000020)	<0.001	0.000130 (0.000019)	<0.001
Log likelihood	-2402.320				-2780.651			
Respondents, n	274				303			
Observations, n	9864				10908			

ASC (opt-out): Alternative Specific Constant for opt-out; SD: Standard Deviation; SE: Standard Error.

**Table 7:** Subgroup analyses: Single child status.

Attribute levels	Single-child family students				Non-single child family students			
	β (SE)	*P*-value	SD (SE)	*P*-value	β (SE)	*P*-value	SD (SE)	*P*-value
**ASC (opt-out)**	5.852(0.321)	<0.001	2.200(0.153)	<0.001	4.283(0.202)	<0.001	1.761(0.148)	<0.001
**Location: Township or rural (ref)**								
County	0.353(0.113)	0.002	0.337(0.177)	0.141	0.456(0.073)	<0.001	0.025(0.234)	0.914
City	1.547(0.146)	<0.001	1.392(0.106)	<0.001	0.757(0.086)	<0.001	0.787(0.101)	<0.001
***Bianzhi*: None (ref)**								
Offer	0.635(0.100)	<0.001	0.850(0.084)	<0.001	0.794(0.074)	<0.001	0.816(0.083)	<0.001
**Training and career development opportunity: Insufficient (ref)**								
Average	-0.065(0.111)	0.563	0.501(0.186)	0.002	0.073(0.076)	0.337	0.388(0.151)	0.010
Sufficient	0.856(0.115)	<0.001	0.674(0.125)	<0.001	0.844(0.082)	<0.001	0.580(0.119)	<0.001
**Working environment: Poor (ref)**								
Common	0.976(0.112)	<0.001	0.134(0.128)	0.397	0.976(0.081)	<0.001	0.029(0.136)	0.829
Superior	0.976(0.109)	<0.001	0.183(0.200)	0.440	1.226(0.080)	<0.001	0.337(0.137)	0.014
**Workload: Heavy (ref)**								
Normal	0.978(0.110)	<0.001	0.101(0.296)	0.700	0.949(0.075)	<0.001	0.053(0.202)	0.795
Light	1.403(0.122)	<0.001	0.326(0.168)	0.107	1.059(0.081)	<0.001	0.023(0.157)	0.882
**Monthly income**	0.000528 (0.000027)	<0.001	0.000117 (0.000029)	<0.001	0.000462 (0.000019)	<0.001	0.000147 (0.000019)	<0.001
Log likelihood	-1940.318				-3256.299			
Respondents, n	220				357			
Observations, n	7920				12852			

ASC (opt-out): Alternative Specific Constant for opt-out; SD: Standard Deviation; SE: Standard Error.

**Table 8:** Subgroup analyses: Universities.

Attribute levels	Eastern university				Middle university				Western university			
	β (SE)	*P*-value	SD (SE)	*P*-value	β (SE)	*P*-value	SD (SE)	*P*-value	β (SE)	*P*-value	SD (SE)	*P*-value
**ASC (opt-out)**	5.077(0.234)	<0.001	2.137(0.149)	<0.001	4.153(0.343)	<0.001	1.921(0.267)	<0.001	4.984(0.405)	<0.001	1.305(0.225)	<0.001
**Location: Township or rural (ref)**												
County	0.456(0.083)	<0.001	0.251(0.211)	0.234	0.257(0.122)	0.035	0.201(0.483)	0.678	0.539(0.153)	<0.001	0.462(0.247)	0.090
City	1.175(0.107)	<0.001	1.206(0.111)	<0.001	0.850(0.148)	<0.001	1.010(0.176)	<0.001	0.862(0.161)	<0.001	0.686(0.203)	<0.001
***Bianzhi*: None (ref)**												
Offer	0.602(0.074)	<0.001	0.674(0.099)	<0.001	0.908(0.143)	<0.001	1.143(0.149)	<0.001	0.905(0.134)	<0.001	0.559(0.185)	0.002
**Training and career development opportunity: Insufficient (ref)**												
Average	0.093(0.084)	0.270	0.474(0.152)	0.002	-0.006(0.127)	0.961	0.490(0.224)	0.028	-0.054(0.141)	0.703	0.003(0.308)	0.991
Sufficient	0.890(0.089)	<0.001	0.629(0.125)	<0.001	0.814(0.136)	<0.001	0.620(0.170)	<0.001	0.750(0.150)	<0.001	0.342(0.295)	0.247
**Working environment: Poor (ref)**												
Common	1.033(0.087)	<0.001	0.009(0.132)	0.947	0.789(0.130)	<0.001	0.002(0.207)	0.992	0.988(0.157)	<0.001	0.172(0.615)	0.707
Superior	1.158(0.086)	<0.001	0.159(0.241)	0.509	1.010(0.127)	<0.001	0.253(0.294)	0.390	1.347(0.157)	<0.001	0.397(0.203)	0.091
**Workload: Heavy (ref)**												
Normal	0.792(0.082)	<0.001	0.037(0.162)	0.821	1.193(0.130)	<0.001	0.168(0.359)	0.640	1.163(0.153)	<0.001	0.003(0.267)	0.993
Light	1.128(0.090)	<0.001	0.042(0.180)	0.815	1.293(0.136)	<0.001	0.253(0.394)	0.520	1.100(0.159)	<0.001	0.106(0.218)	0.637
**Monthly income**	0.000510 (0.000021)	<0.001	0.000987 (0.000023)	<0.001	0.000436 (0.000033)	<0.001	0.000181 (0.000034)	<0.001	0.000469 (0.000038)	<0.001	0.000151 (0.000036)	<0.001
Log likelihood	-2957.892				-1322.383				-917.927			
Respondents, n	332				145				100			
Observations, n	11952				5220				3600			

ASC (opt-out): Alternative Specific Constant for opt-out; SD: Standard Deviation; SE: Standard Error.

**Table 9:** WTP (CNY) for subgroups; 95% CI.

Attribute levels	Urban background	Rural or county background	Annual family income: > 50000 CNY	Annual family income: ≤ 5000 CNY	Single-child family students	Non-single child family students	Eastern university	Middle university	Western university
**Location: County**	892.834 (366.012, 1448.921)	885.565 (592.662, 1185.933)	1171.308 (756.556, 1609.970)	661.442 (332.843, 996.318)	668.374 (255.925, 1107.591)	987.044 (682.144, 1311.455)	895.512 (574.838, 1228.500)	589.134 (46.071, 1161.258)	1149.951 (520.763, 1832.550)
**Location: City**	4099.869 (3484.536, 4757.348)	1202.488 (876.651, 1528.612)	3349.713 (2861.935, 3859.745)	1123.474 (742.524, 1503.140)	2929.250 (2406.495, 3492.587)	1638.315 (1274.007, 2013.219)	2306.054 (1899.230, 2728.549)	1950.580 (1287.243, 2656.825)	1838.275 (1162.746, 2571.750)
***Bianzhi*: Offer**	1153.743 (737.485, 1582.803)	1678.255 (1407.950, 1960.301)	1484.337 (1140.866, 1841.557)	1488.486 (1180.687, 1807.910)	1202.974 (847.018, 1579.977)	1718.676 (1416.351, 2035.973)	1181.644 (906.312, 1459.830)	2084.123 (1478.953, 2717.309)	1930.201 (1408.975, 2477.270)
**Career development opportunity: Average**	-115.792 (-605.211, 369.514)	87.878 (-215.939, 388.283)	111.495 (-293.083, 517.152)	69.209 (-266.788, 404.180)	-122.176 (-546.528, 293.924)	158.069 (-168.504, 489.671)	182.850 (-143.576, 513.375)	- 14.233 (-585.652, 578.738)	-114.834 (-695.911, 501.266)
**Career development opportunity: Sufficient**	1545.169 (1083.161, 2038.920)	1796.990 (1469.150, 2141.763)	2107.101 (1702.911, 2534.917)	1482.476 (1118.502, 1865.003)	1621.074 (1186.212, 2075.235)	1828.093 (1471.906, 2198.849)	1746.502 (1394.133, 2114.573)	1868.966 (1240.943, 2557.227)	1599.159 (965.616, 2298.128)
**Working environment: Common**	1949.869 (1459.079, 2458.366)	2107.493 (1794.297, 2431.551)	2031.702 (1632.044, 2444.785)	1947.257 (1597.662, 2305.731)	1846.856 (1439.783, 2280.083)	2113.806 (1781.364, 2462.648)	2028.211 (1703.647, 2372.020)	1811.847 (1238.822, 2440.163)	2106.743 (1474.546, 2799.193)
**Working environment: Superior**	2290.657 (1808.844, 2792.515)	2387.833 (2067.085, 2713.282)	2169.596 (1763.033, 2587.732)	2538.689 (2187.152, 2900.917)	1848.529 (1439.783, 2280.083)	2654.387 (2314.435, 3023.983)	2272.082 (1944.230, 2626.915)	2318.090 (1757.441, 2952.760)	2873.652 (2232.071, 3621.463)
**Workload: Normal**	1019.687 (578.808, 1474.667)	2402.362 (2102.617, 2718.347)	1839.739 (1469.526, 2226.138)	2120.961 (1792.102, 2466.155)	1851.058 (1452.318, 2271.303)	2055.134 (1740.963, 2386.043)	1554.878 (1250.765, 1878.817)	2738.298 (2172.373, 3391.926)	2480.473 (1888.392, 3143.388)
**Workload: Light**	2115.990 (1653.249, 2596.132)	2527.595 (2223.830, 2845.693)	2525.172 (2137.248, 2934.035)	2354.556 (2013.332, 2707.237)	2656.212 (2244.462, 3090.827)	2294.297 (1968.714, 2634.686)	2214.622 (1890.920, 2542.078)	2967.878 (2386.470, 3605.209)	2347.219 (1721.009, 3004.588)

US$1 = CNY 6.759; 95% CI = 95% confidence interval.

### Simulated preferences for job posting under various potential policy scenarios

**Fig 1:**
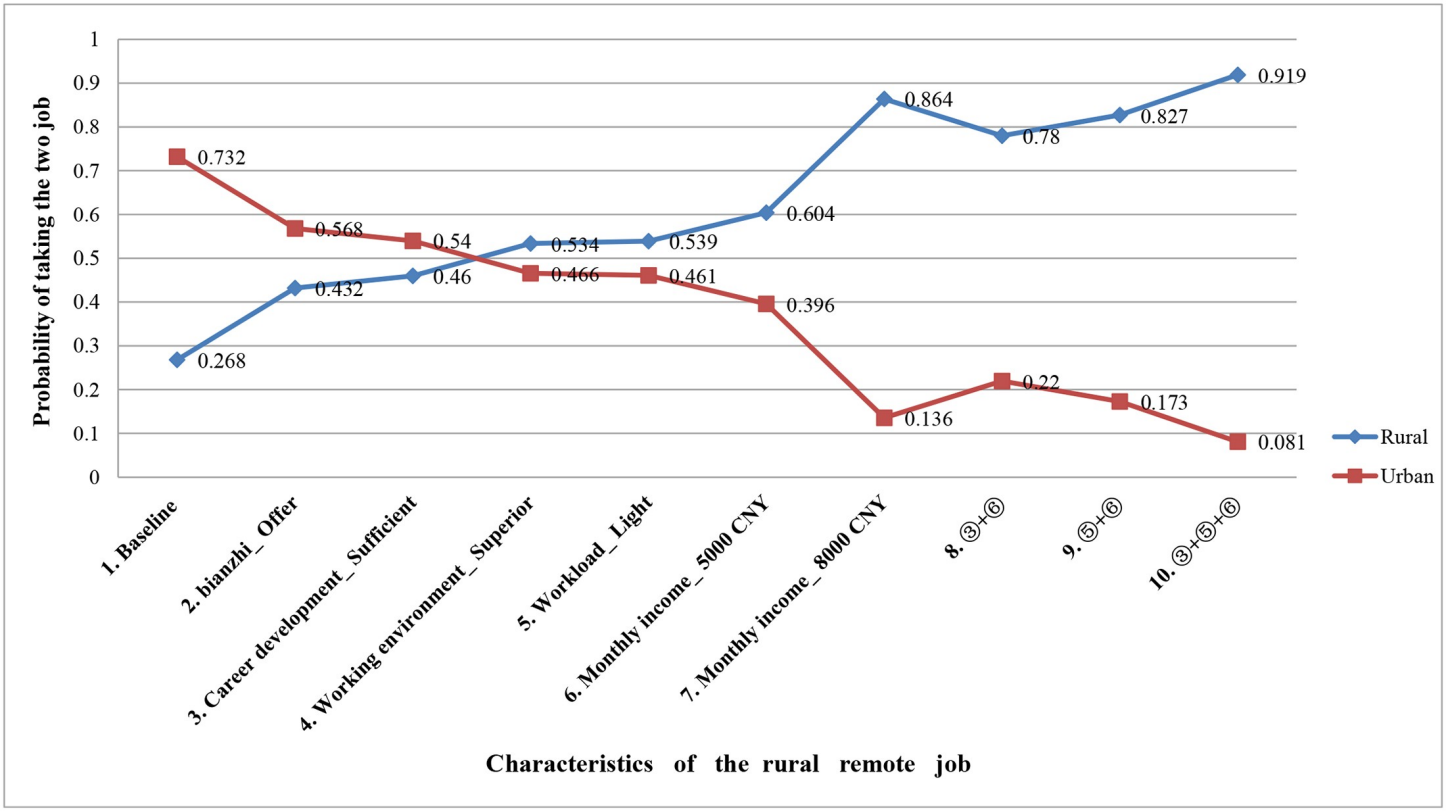
Simulated preferences for job posting under various potential policy scenarios. Changes in the probabilities of taking a job, rural remote versus urban, as conditions in the rural remote job improve.

Fig 1 shows the varying probabilities of taking a rural remote job versus one in the city, with various job conditions. The initial (baseline: 2000 CNY monthly income; heavy workload; poor working environment; insufficient training and career development opportunity; no *bianzhi*) probability of taking the rural remote job is 0.268, hence the probability of taking the job in the city is 0.732. The job in the city is thus preferred. For the single incentives, if superior working environment was provided for the rural remote job, the probability of taking that job will increases to 0.534 (so the remote job is preferred). For the selective multiple incentives, the policy “③+⑤+⑥”was the most attractive one, as it can increase the probability of taking rural job to 0.919.

## Discussion

To the best of our knowledge, this is the first study using DCE methodology to investigate job preferences of healthcare administration students internationally. All six attributes considered in our study were found to be statistically significant in influencing student preferences to choose a job.

Overall, monthly income and workload were the two strongest drivers of choice. This finding is consistent with the results of an earlier quantitative study in which lower income and higher workload are the two major contributing factors toward job dissatisfaction in China [53]. Other study conducted with public health undergraduate graduates in China further found the most frequently cited factors for their lack of commitment to public health facilities after graduation were associated with remuneration, geographic locations, and career advancement [15]. For the single incentives, increasing the monthly income from 2000 to 8000 CNY had the largest effect on preference for rural postings. However, increasing monthly income alone may not be the most efficient way to recruit students to rural areas. The simulation results in our study shows that although raising monthly income from 2000 to 5000 CNY can increases the probability of taking a rural remote job by 0.336, increasing it by another 3000 to 8000 CNY increases the probability by only 0.260. Furthermore, although higher salary has a large effect on preference for rural positions, it is often not possible in the real world, for it may require significant financial investments upfront, such that policy-makers may be deterred from implementing this intervention [6]. Alternatively, a combination of non-monetary incentives (such as superior working environment, sufficient training and career development opportunities) or non-monetary combined with monetary incentives (such as light workload with 5000 CNY per month) can achieve similar impact as the highest monetary incentive. It may thus be wise, after raising monthly income to a certain level, to focus on other types of policies.

Among non-monetary attributes, workload is one of the most important factors, especially for students from western and middle universities. Healthcare administration is undergoing the transition from experiential management to scientific management in China [12]. To promote the development of health service and achieve the ultimate goals of the healthcare reform, healthcare administrators are required to keep learning the new theory and knowledge of health service management, and try their best to ameliorate themselves during the study and practice [12]. Undoubtedly, this will increase the health workers’ workloads. As one of the major sources of job stress, heavy workloads will hinder health workers from taking jobs in rural areas, and in turn, staff shortages will impact negatively on the motivation of the existing staff as they have to deal with increased workload [54, 55]. Other studies also found that increasing the number of health workers can diminish the reasons for non-retention in rural and remote areas, such as high workload [56–58].

In terms of the working environment, it has been pointed out that the shortage of health workers in rural and remote areas is more significantly affected by the problem of retention rather than of recruitment [3]. Because rural and remote health facilities are often poorly equipped and inadequately supplied with drugs, the physical working conditions are severe, and staff are poorly supported or supervised and often feel isolated and neglected [33]. Working environment in our study refers to management support, the relationship between superior and subordinate, high-risk work environments and availability of equipment. The in-depth interview with sixteen healthcare administration students conducted before the DCE provided us with some insights on the importance of working environment. During the interviews, most students indicated that good relationships with colleagues and support from superiors and subordinates can help to improve job satisfaction. The main effects model results further demonstrated students were willing to pay 2398 CNY to obtain a job position with superior rather than poor working environments which is almost equal to a light workload.

In general, healthcare administration students prefer to work in the city rather than rural areas. The initial probability of taking the rural job is 0.268, while 0.732 of taking the urban job. Good living conditions are essential to influence worker decisions to move and stay in a particular area [33]. However, living conditions in most of the rural areas in China are still poor compared with urban areas in terms of infrastructure (e.g. telecommunications and transportation), schools for children and employment opportunities for spouse. To address those problems, coordinated actions should be taken by China’s government, as they are linked to the wider socio-economic and political context. In addition, preferences of location may also depend on what kind of living conditions health personnel are used to. Our study reveals that those participants who have an urban background and/or with higher family incomes were willing to pay more on working in the city. Other studies conducted in high and low-income countries [59–61] also found that a rural upbringing can increase the chances of health workers returning to practice in rural remote areas [62, 63]. Therefore, attracting and retaining healthcare administration students with a rural background for rural areas would be a more efficient strategy.

The impact of opportunities for training and career development has been shown continually throughout previous studies, and is usually one of the most important factors underlying career choice [27, 64–66]. However, although career development was valued in our study, it did not appear to be as important as the workload or working environment. It was also remarkably homogeneous in terms of how this attribute was valued in a health-related job choice by different participants. It could be that the respondents in our study were still final year healthcare administration students just finishing their specialty placement and further training opportunities may not be regarded as important at the very beginning of their career.

An unexpected finding from our study is the relatively lower utility of *bianzhi* in job preferences. It was contrary to another study on medical workers which was strongly suggestive of a preference for providing *bianzhi* in China [41]. There could be two explanations. Firstly, all participants in our study were born after 1990. For this younger generation a job with *bianzhi* may be more stable but may not be as important as it may be for older generations. Secondly, the recent health reform has witnessed an important role of the private health sector in the Chinese health system. Healthcare administration students have more opportunities to work in private sectors which normally provide better salaries and working environments. In a previous study conducted by our team, we found similar results for undergraduate medical students [24]. Consequently, the *bianzhi* in the health sector may not be as important as it used to be. In addition, students from western and middle universities valued *bianzhi* higher than students from eastern universities. It could be explained by the different socio-economic status and educational styles between eastern, middle and western areas. It should be considered by policy-makers that offering *bianzhi* to students from western and middle universities would be more efficient for the recruitment and retention of them to rural and remote health institutions.

This study had several limitations. Firstly, although several quality control procedures had been adopted in the data collection, there were still 69 (10.7%) participants among all respondents failed the internal consistency test. That may due to high cognitive burden, or some students were less motivated to response to the hypothetical experimental scenarios. Secondly, any inferences made on the basis of these results apply only to healthcare administration students, not to healthcare administrators. These two groups may differ in their preferences for job position, suggesting the need for further investigation. Thirdly, there could be some concern that given the birth control policy in China during the past 20 years, the proportion of respondents who are the single child of their family (38%) may seem to be low in our study. This may be owing to the fact that most students come from rural areas, whilst only one third come from urban areas. According to a study conducted by Huang RL [67], 74% of single-child families are concentrated in urban areas, while only 26% concentrated in rural areas in China. Fourthly, only eight universities from north of China were included in this study, further research will be needed to clarify whether there exists difference in job preferences between the north and south healthcare administration students of China.

## Conclusions

In conclusion, for healthcare administration students in China, the preferred scenario was to select a better working environment job with light workload located in the city, which can offer 8000 CNY monthly, sufficient training and career development opportunities and with *bianzhi*. Both monetary and non-monetary attributes were found to be significantly influential in affecting students’ preferences for choosing a job. In addition, there exists a certain degree of both observable and unobservable preference heterogeneity among students, which should also be taken into account in developing more effective policy incentive packages.

## Supporting information

S1 Fig An example of discrete choice experiment in Chinese. (TIF)

S1 File Questionnaires and DCE data from participants in this study. (ZIP)

S1 Table Mixed logit estimates (n = 646). (DOCX)

S2 Table Conditional logit estimates (n = 577). (DOCX)
