# Supplementary figures and images for: Job preferences for healthcare administration students in China: A discrete choice experiment
Source: PLoS One. 2019 Jan 25;14(1):e0211345. doi: 10.1371/journal.pone.0211345 (PMC6347231; doi:10.1371/journal.pone.0211345)

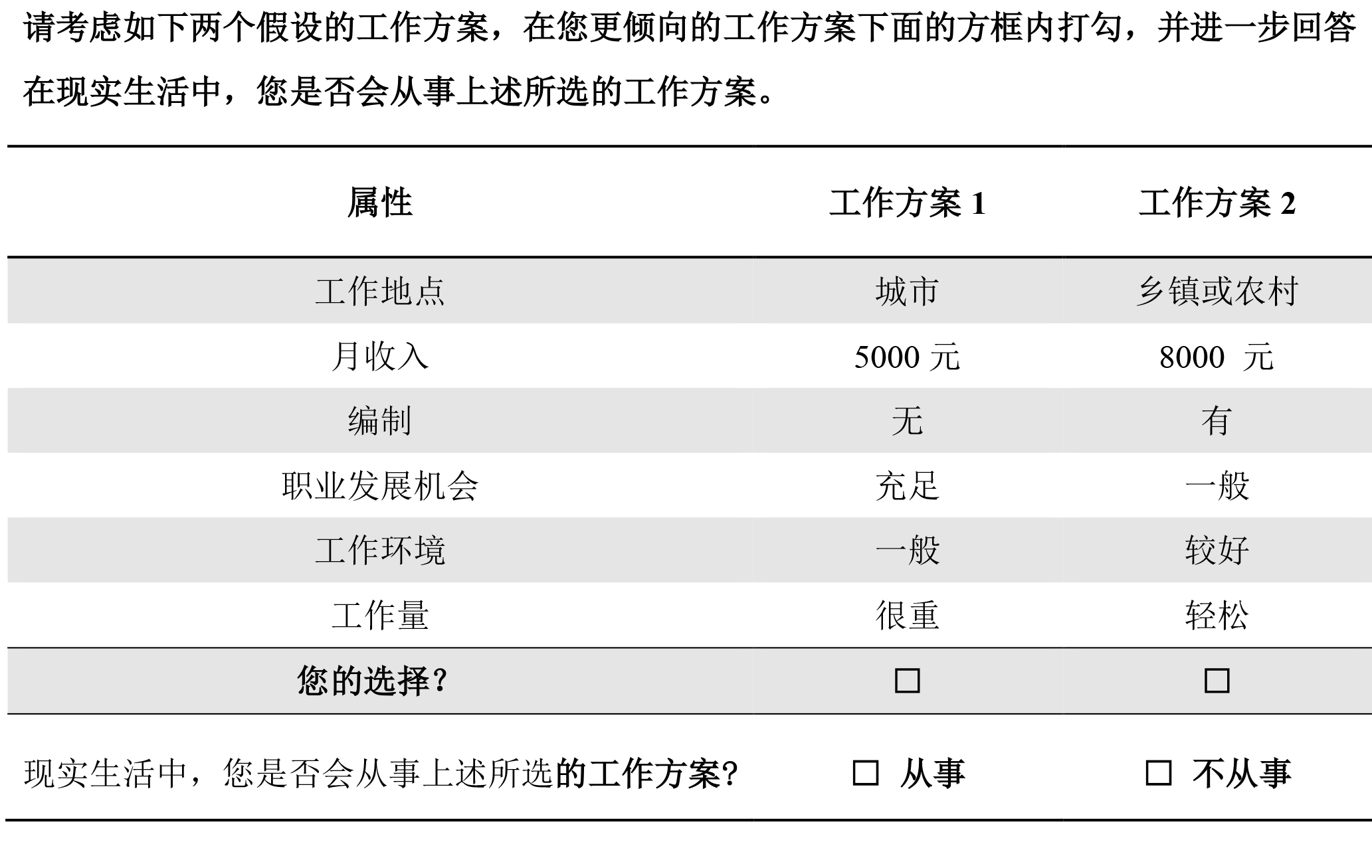

Supplement: S1 Fig — (TIF) [file pone.0211345.s001.tif]
