# Supplementary material for: Job preferences for healthcare administration students in China: A discrete choice experiment
Source: PLoS One. 2019 Jan 25;14(1):e0211345. doi: 10.1371/journal.pone.0211345 (PMC6347231; doi:10.1371/journal.pone.0211345)
Supplement: S1 File — (ZIP) [file pone.0211345.s002.zip › Supporting Information/Questionnaireú║Version 2.docx]

**Part 1: Participants’ socio-demographic characteristics**

***1. Age: ____***

***2. Gender:***

A: Male B: Female

***3. Birthplace:***

A: Urban B: County C: Rural or village

***4. Single child:***

A: Yes B: No

***5. Monthly consumption (CNY):***

A: < 800 B: 800 - 1500 C: 1500 - 2500 D: > 2500

***6. Annual family income (CNY):***

A: < 30,000 B: 30,000 - 50,000 C: 50,000 - 70,000 D: > 70,000

***7. Career planning?***

A: Health related work B: Further study C: Starting a Business D: Others

***8. Please rank the attributes from most important to least important with respect to your preferences for choosing a job?***

A: Location B: Monthly income C: *Bianzhi* D: 3 Training and career development opportunity E: Working environment F: Workload

**Part 2: The questionnaire of discrete choice experiments (Version 1)**

**Project 1**

| Attribute | Screening Scenario 1 | Screening Scenario 2 |
| --- | --- | --- |
| Location | Township or rural | County |
| Monthly income | 2000 CNY | 5000 CNY |
| *bianzhi* | Offer | None |
| Training and career development opportunity | Average | Sufficient |
| Working environment | Common | Superior |
| Workload | Light | Heavy |
| Which of these jobs would you prefer? | ☐ | ☐ |
| Will you actually take up the job you chose if it was offered to you? | ☐ Yes | ☐ No |

**Project 2**

| Attribute | Screening Scenario 1 | Screening Scenario 2 |
| --- | --- | --- |
| Location | City | Township or rural |
| Monthly income | 5000 CNY | 2000 CNY |
| *bianzhi* | None | Offer |
| Training and career development opportunity | Sufficient | Insufficient |
| Working environment | Poor | Common |
| Workload | Light | Heavy |
| Which of these jobs would you prefer? | ☐ | ☐ |
| Will you actually take up the job you chose if it was offered to you? | ☐ Yes | ☐ No |

**Project 3**

| Attribute | Screening Scenario 1 | Screening Scenario 2 |
| --- | --- | --- |
| Location | Township or rural | City |
| Monthly income | 5000 CNY | 8000 CNY |
| *bianzhi* | Offer | None |
| Training and career development opportunity | Average | Insufficient |
| Working environment | Common | Poor |
| Workload | Normal | Heavy |
| Which of these jobs would you prefer? | ☐ | ☐ |
| Will you actually take up the job you chose if it was offered to you? | ☐ Yes | ☐ No |

**Project 4**

| Attribute | Screening Scenario 1 | Screening Scenario 2 |
| --- | --- | --- |
| Location | County | City |
| Monthly income | 2000 CNY | 8000 CNY |
| *bianzhi* | None | Offer |
| Training and career development opportunity | Average | Insufficient |
| Working environment | Superior | Common |
| Workload | Heavy | Normal |
| Which of these jobs would you prefer? | ☐ | ☐ |
| Will you actually take up the job you chose if it was offered to you? | ☐ Yes | ☐ No |

**Project 5**

| Attribute | Screening Scenario 1 | Screening Scenario 2 |
| --- | --- | --- |
| Location | City | County |
| Monthly income | 8000 CNY | 5000 CNY |
| *bianzhi* | None | Offer |
| Training and career development opportunity | Sufficient | Insufficient |
| Working environment | Superior | Poor |
| Workload | Heavy | Light |
| Which of these jobs would you prefer? | ☐ | ☐ |
| Will you actually take up the job you chose if it was offered to you? | ☐ Yes | ☐ No |

**Project 6**

| Attribute | Screening Scenario 1 | Screening Scenario 2 |
| --- | --- | --- |
| Location | City | County |
| Monthly income | 5000 CNY | 2000 CNY |
| *bianzhi* | Offer | None |
| Training and career development opportunity | Sufficient | Average |
| Working environment | Superior | Poor |
| Workload | Light | Normal |
| Which of these jobs would you prefer? | ☐ | ☐ |
| Will you actually take up the job you chose if it was offered to you? | ☐ Yes | ☐ No |

**Project 7**

| Attribute | Screening Scenario 1 | Screening Scenario 2 |
| --- | --- | --- |
| Location | City | County |
| Monthly income | 5000 CNY | 8000 CNY |
| *bianzhi* | None | Offer |
| Training and career development opportunity | Average | Sufficient |
| Working environment | Poor | Common |
| Workload | Light | Normal |
| Which of these jobs would you prefer? | ☐ | ☐ |
| Will you actually take up the job you chose if it was offered to you? | ☐ Yes | ☐ No |

**Project 8**

| Attribute | Screening Scenario 1 | Screening Scenario 2 |
| --- | --- | --- |
| Location | City | Township or rural |
| Monthly income | 5000 CNY | 8000 CNY |
| *bianzhi* | None | Offer |
| Training and career development opportunity | Average | Sufficient |
| Working environment | Common | Superior |
| Workload | Normal | Light |
| Which of these jobs would you prefer? | ☐ | ☐ |
| Will you actually take up the job you chose if it was offered to you? | ☐ Yes | ☐ No |

**Project 9**

| Attribute | Screening Scenario 1 | Screening Scenario 2 |
| --- | --- | --- |
| Location | Township or rural | County |
| Monthly income | 8000 CNY | 2000 CNY |
| *bianzhi* | Offer | None |
| Training and career development opportunity | Average | Insufficient |
| Working environment | Poor | Superior |
| Workload | Heavy | Light |
| Which of these jobs would you prefer? | ☐ | ☐ |
| Will you actually take up the job you chose if it was offered to you? | ☐ Yes | ☐ No |

**Project 10**

| Attribute | Screening Scenario 1 | Screening Scenario 2 |
| --- | --- | --- |
| Location | County | Township or rural |
| Monthly income | 5000 CNY | 8000 CNY |
| *bianzhi* | Offer | None |
| Training and career development opportunity | Average | Sufficient |
| Working environment | Common | Poor |
| Workload | Heavy | Normal |
| Which of these jobs would you prefer? | ☐ | ☐ |
| Will you actually take up the job you chose if it was offered to you? | ☐ Yes | ☐ No |

**Project 11**

| Attribute | Screening Scenario 1 | Screening Scenario 2 |
| --- | --- | --- |
| Location | Township or rural | City |
| Monthly income | 5000 CNY | 2000 CNY |
| *bianzhi* | None | Offer |
| Training and career development opportunity | Insufficient | Average |
| Working environment | Poor | Superior |
| Workload | Heavy | Light |
| Which of these jobs would you prefer? | ☐ | ☐ |
| Will you actually take up the job you chose if it was offered to you? | ☐ Yes | ☐ No |

**Project 12**

| Attribute | Screening Scenario 1 | Screening Scenario 2 |
| --- | --- | --- |
| Location | Township or rural | County |
| Monthly income | 5000 CNY | 8000 CNY |
| *bianzhi* | None | Offer |
| Training and career development opportunity | Insufficient | Average |
| Working environment | Superior | Common |
| Workload | Normal | Heavy |
| Which of these jobs would you prefer? | ☐ | ☐ |
| Will you actually take up the job you chose if it was offered to you? | ☐ Yes | ☐ No |

**Project 13**

| Attribute | Screening Scenario 1 | Screening Scenario 2 |
| --- | --- | --- |
| Location | Township or rural | City |
| Monthly income | 5000 CNY | 8000 CNY |
| *bianzhi* | Offer | None |
| Training and career development opportunity | Average | Insufficient |
| Working environment | Common | Poor |
| Workload | Normal | Heavy |
| Which of these jobs would you prefer? | ☐ | ☐ |
| Will you actually take up the job you chose if it was offered to you? | ☐ Yes | ☐ No |
