# Supplementary material for: Job preferences for healthcare administration students in China: A discrete choice experiment
Source: PLoS One. 2019 Jan 25;14(1):e0211345. doi: 10.1371/journal.pone.0211345 (PMC6347231; doi:10.1371/journal.pone.0211345)
Supplement: S1 Table — (DOCX) [file pone.0211345.s003.docx]

**S1 Table. Mixed logit estimates (n=646).**

| **Attribute levels** |  | **β (SE)** | ***P*-value** |  | **SD (SE)** | ***P*-value** |
| --- | --- | --- | --- | --- | --- | --- |
| **ASC** **(opt-out)** |  | 4.559(0.156) | < 0.001 |  | 1.898(0.097) | < 0.001 |
| **Location: Township or Rural (ref)** |  |  |  |  |  |  |
| County |  | 0.348(0.057) | < 0.001 |  | 0.198(0.175) | 0.259 |
| City |  | 0.928(0.069) | < 0.001 |  | 0.983(0.077) | < 0.001 |
| ***Bianzhi:* None (ref)** |  |  |  |  |  |  |
| Offer |  | 0.641(0.054) | < 0.001 |  | 0.788(0.064) | < 0.001 |
| **Training and career development: Insufficient (ref)** | | | |  |  |  |
| Average |  | 0.084(0.058) | 0.144 |  | 0.380(0.121) | 0.002 |
| Sufficient |  | 0.909(0.062) | < 0.001 |  | 0.629(0.086) | < 0.001 |
| **Work environment: Poor (ref)** |  |  |  |  |  |  |
| Common |  | 0.928(0.060) | < 0.001 |  | 0.040(0.103) | 0.701 |
| Superior |  | 1.148(0.059) | < 0.001 |  | 0.169(0.210) | 0.420 |
| **Workload: Heavy (ref)** |  |  |  |  |  |  |
| Normal |  | 0.859(0.057) | < 0.001 |  | 0.035(0.209) | 0.865 |
| Light |  | 1.067(0.062) | < 0.001 |  | 0.039(0.126) | 0.757 |
| **Monthly income** |  | 0.000444(0.000014) | < 0.001 |  | 0.000140(0.000141) | < 0.001 |
| Log likelihood |  | -6000.084  646  23256 | | | | |
| Respondents, n |  |  |  |  |  |  |
| Observations, n |  |  |  |  |  |  |

β: The coefficients (β) represents the mean relative utility of each attribute conditional on other attributes in a choice set where larger values indicate greater utility and more preferred attributes; ASC (opt-out): Alternative Specific Constant for opt-out; SD: Standard Deviation estimates reflect preference heterogeneity in the students, a possible indication of unmeasured factors influencing the strength and direction of preference; 95% CI = 95% Confidence Interval; SE: Standard Error.
