# Supplementary material for: Job preferences for healthcare administration students in China: A discrete choice experiment
Source: PLoS One. 2019 Jan 25;14(1):e0211345. doi: 10.1371/journal.pone.0211345 (PMC6347231; doi:10.1371/journal.pone.0211345)
Supplement: S2 Table — (DOCX) [file pone.0211345.s004.docx]

**S2 Table. Conditional logit estimates (n=577).**

| **Attribute levels** | **β (SE)** | ***P*-value** |
| --- | --- | --- |
| **ASC** **(opt-out)** | 3.775(0.132) | < 0.001 |
| **Location: Township or Rural (ref)** |  |  |
| County | 0.271(0.050) | < 0.001 |
| City | 0.758(0.062) | < 0.001 |
| ***Bianzhi:* None (ref)** |  |  |
| Offer | 0.555(0.045) | < 0.001 |
| **Training and career development: Insufficient (ref)** | |  |
| Average | 0.106(0.047) | 0.025 |
| Sufficient | 0.623(0.049) | < 0.001 |
| **Work environment: Poor (ref)** |  |  |
| Common | 0.639(0.050) | < 0.001 |
| Superior | 0.822(0.052) | < 0.001 |
| **Workload: Heavy (ref)** |  |  |
| Normal | 0.712(0.053) | < 0.001 |
| Light | 0.846(0.057) | < 0.001 |
| **Monthly income** | 0.000350(0.000011) | < 0.001 |
| AIC | 12022.63 | |
| BIC | 12109.98 | |
| Log likelihood | -6000.315 | |
| Respondents, n | 577 | |
| Observations, n | 20772 | |

β: The coefficients (β) represents the mean relative utility of each attribute conditional on other attributes in a choice set where larger values indicate greater utility and more preferred attributes; ASC (opt-out): Alternative Specific Constant for opt-out; AIC: Akaike Information Criterion; AIC: Akaike Information Criterion; BIC: Bayesian Information Criterion; SD: Standard Deviation estimates reflect preference heterogeneity in the students, a possible indication of unmeasured factors influencing the strength and direction of preference; 95% CI = 95% Confidence Interval; SE: Standard Error.
